# Twelve-Month Estimated Glomerular Filtration Rate Trajectory at the Time of Kidney Replacement Therapy Counseling Predicts Dialysis Initiation Within One Year

**DOI:** 10.3390/jcm14061981

**Published:** 2025-03-14

**Authors:** Panuwat Chuemor, Kittipan Rerkasem, Apichat Tantraworasin, Jiraporn Khorana, Theerachai Thammathiwat, Watchara Pichitsiri

**Affiliations:** 1 Department of Surgery, Faculty of Medicine, Naresuan University, Phitsanulok 65000, Thailand; chuemor35707@gmail.com; 2 Department of Surgery and Clinical Surgical Research Centre, Faculty of Medicine, Chiangmai University, Chiangmai 50200, Thailand; ohm_med@hotmail.com (A.T.); nanji22@gmail.com (J.K.); 3 Division of Nephrology, Department of Medicine, Faculty of Medicine, Naresuan University, Phitsanulok 65000, Thailand; theerachait@nu.ac.th

**Keywords:** chronic kidney disease, estimated glomerular filtration rate decline, predictive model

## Abstract

**Background/Objectives**: Planned kidney replacement therapy (KRT) proactively selects and prepares appropriate dialysis modalities and ensures timely vascular access—be it arteriovenous or peritoneal—before dialysis is needed. This approach leads to better patient outcomes and fewer complications. We aimed to develop a predictive model using past estimated glomerular filtration rate (eGFR) measurements prior to KRT counseling to estimate individual patients’ likelihood of initiating dialysis. **Methods:** In this prognostic prediction study, we retrospectively analyzed data from chronic kidney disease patients who received KRT counseling at Naresuan University Hospital in Thailand. A logistic regression model was developed incorporating the historical eGFR decline over the preceding twelve months (eGFRr) at the time of counseling. The model’s performance was compared to the predictive accuracy of using a single eGFR measurement, as commonly practiced in clinical settings. **Results:** This study included 103 patients who received their first KRT counseling between 1 January 2016 and 31 December 2022. Within one year, 62% initiated their first dialysis session. The eGFRr was a significant predictor of dialysis initiation. Logistic regression identified six key predictors: past eGFRr, age, systolic blood pressure, primary cause of chronic kidney disease, body mass index categories, and serum calcium levels. The predictive model showed good discriminative ability, with an area under the receiver operating characteristic curve of 0.836 (95% CI 0.754–0.918). **Conclusions:** Our predictive model estimates the likelihood of dialysis initiation, offering valuable decision support insights. Clinical implementation could enhance timely referral and preparation for patients requiring KRT. Prospective validation is needed to confirm its accuracy before clinical use.

## 1. Introduction

Planned kidney replacement therapy (KRT) refers to the modality selected and prepared in advance of the need for dialysis, with vascular access established prior to the initiation of hemodialysis. Comprehensive education and decision aids are crucial in facilitating an informed understanding of KRT among patients with chronic kidney disease (CKD). Effective educational interventions are typically implemented when patients reach CKD stage G4, defined by an estimated glomerular filtration rate (eGFR) of less than 30 mL/min/1.73 m^2^ [1]. The selection of an appropriate dialysis modality should be guided by timely, shared decision-making processes involving the healthcare team, patients, and their family members.

The majority of patients with stage 4 CKD will not progress to end-stage kidney disease (ESKD). Data indicate that only 19.9% of stage 4 CKD patients required KRT over a five-year observation period [2]. Current clinical guidelines do not endorse the pre-emptive initiation of dialysis [3,4]. Although the optimal timing for the initiation of dialysis remains a subject of ongoing debate, the reasons for initiating dialysis in clinical practice are diverse and context-dependent [5]. The preparatory process for KRT is resource-intensive and demands considerable healthcare utilization. Consequently, preparing all CKD G4 patients for dialysis may strain already limited healthcare resources and potentially induce undue anxiety among patients.

Moreover, the timing of pre-dialysis assessments is influenced by several factors, including the degree of kidney function decline, the presence of uremic symptoms, associated comorbidities, and the occurrence of metabolic and hematologic disturbances. These assessments are generally conducted on a monthly or thrice-monthly basis. In patients exhibiting rapid declines in eGFR, more frequent monitoring is necessary to detect uremic symptoms, oliguria, and acid–base or electrolyte imbalances, to adjust diuretics, as well as to address issues related to volume status control [1]. Timing for vascular access—whether arteriovenous or peritoneal dialysis (PD) catheter placement—is crucial and must align with the anticipated start of KRT [6]. According to the 2019 KDOQI clinical practice guidelines for vascular access, a PD catheter should be placed at least two weeks before starting PD, whereas an arteriovenous fistula or graft should be placed approximately 6–9 months before initiating HD [6]. Appropriate dialysis access preparation, following shared decision-making during advanced CKD counseling, is essential for optimal vascular access planning and timely KRT initiation.

The likelihood of initiating dialysis plays a pivotal role in determining the appropriate preparation strategies and intervals for pre-dialysis follow-up. The use of eGFR-based predictive models may prove valuable in identifying subgroups of patients who are more likely to require dialysis and in predicting when dialysis initiation may become necessary. Patients within these subgroups should receive intensive counseling regarding potential complications, prognosis, available treatment options for advancing disease, and the decision-making process for selecting the appropriate modality of KRT.

## 2. Materials and Methods

### 2.1. Study Design and Setting

The retrospective cohort study involved reviewing data from patients diagnosed with chronic kidney disease who underwent KRT counseling at Naresuan University Hospital.

### 2.2. Study Patients and Data Collection

Patients who engaged in KRT counseling between 1 January 2016 and 31 December 2022, were included in this study. Exclusion criteria included individuals diagnosed with acute illness or acute kidney injury who did not recover to their baseline eGFR, as well as those with hemodynamic instability at KRT initiation, severe infection, or advanced malignancy. This study was conducted according to the guidelines of the Declaration of Helsinki and was approved by the Institutional Review Board of Naresuan University (IRB No. P3-0018/2568, protocol code 044/2025) on 13 February 2025. Enrollment took place on the day of KRT counseling, with baseline covariates recorded at the time of enrollment, which served as the cohort’s entry point (T0).

### 2.3. Candidate Predictors

In this study, eGFR was calculated using the CKD-EPI formula, with measurements documented during regular nephrology care visits every two months. The primary focus of the analysis was on eGFRr, representing the rate of change in eGFR over the 12 months preceding the first KRT counseling session. Baseline characteristics were collected to compare early and no KRT initiation based on eGFRr over one year. These included age, gender, body mass index (BMI), systolic blood pressure, primary cause of CKD, history of recent cardiovascular events, eGFRi, eGFR at the time of the first dialysis session, hemoglobin level, serum albumin, serum calcium, serum phosphate, and the use of renin–angiotensin–aldosterone system inhibitors (RAASi).

### 2.4. Clinical Endpoint Definition

In our institution, the decision to initiate the first dialysis session is based on the nephrologist’s clinical assessment. This assessment aligns with a diagnosis of CKD G5 with an eGFR below 15 mL/min/1.73 m^2^ and considers factors such as the presence of uremic symptoms, inadequate response to pharmacological management of volume overload or metabolic disturbances, or an asymptomatic eGFR below 6 mL/min/1.73 m^2^ [7].

### 2.5. Statistical Analysis

Statistical analyses used Stata version 17 (StataCorp LLC, College Station, TX, USA). Descriptive statistics summarize baseline characteristics, presenting mean and SD or median and interquartile range for continuous variables and frequency/percentages for categorical variables. Continuous variable comparisons employed *t*-tests or rank-sum tests, while categorical variables were assessed using an exact probability test. To quantify the rate of decline in eGFR, we calculated the slope of the linear regression using the eGFR measurements collected at 2-month intervals over the 12-month period before KRT counseling. Factors influencing eGFRr over the preceding twelve months were assessed using Gaussian regression. This analysis included age, gender, systolic blood pressure classification, cause of CKD, recent major cardiovascular events, hypoalbuminemia, serum calcium levels, hyperphosphatemia at the time of KRT counseling, and recent use of renin–angiotensin–aldosterone system inhibitors (RAASi) as covariates. Potential predictors were primarily selected, including eGFRi and eGFRr, along with relevant clinical parameters assessed at the time of KRT counseling for incorporation into the predictive model. Given that some patients may die following KRT counseling, death was considered a competing event prior to the initiation of dialysis. To examine the potential predictors for the early initiation of dialysis within one year of KRT counseling, we employed competing risks time-to-event analysis. In cases where the proportional hazards assumption was violated, a logistic regression model was employed to estimate the probability of dialysis initiation. This model incorporated several covariates: estimated glomerular filtration rate (eGFRr), age, body mass index (BMI) category, systolic blood pressure, primary cause of chronic kidney disease (CKD), and serum calcium level. Following TRIPOD guidelines, continuous predictors, such as eGFRr and eGFRi, were treated as continuous variables. Locally weighted scatterplot smoothing (LOWESS) and quadratic and fractional polynomial plots were utilized to investigate potential non-linear relationships between predictors and outcomes [8].

### 2.6. Clinical Implication

To enhance practical applicability, the predictive model has been integrated into a web application. By inputting six parameters, including eGFRr, the application provides an estimation of the probability that a patient will require dialysis within two years. The application subsequently offers personalized recommendations for clinical management tailored to each patient’s individual risk profile. For patients with a high probability of requiring dialysis (greater than 80%), we recommend urgent referral for further assessment, preparation for vascular access, and more frequent monitoring for the development of uremic symptoms. In contrast, for patients with a lower probability (less than 80%), we advise regular follow-up to monitor symptoms and re-assessment using the predictive model as needed.

### 2.7. Study Size Considerations

In developing our prognostic predictive model, we selected six variables for inclusion in the logistic regression analysis, as outlined previously. To ensure compliance with established guidelines, it is recommended that there be at least 10 to 15 clinical endpoint events for each predictor variable incorporated into the model, in accordance with the transparent reporting of a multivariable prediction model for individual prognosis or diagnosis (TRIPOD) guidelines. The binary outcome in our study is defined as the initiation of dialysis within one year following KRT counseling. Accordingly, to accurately distinguish between patients who will initiate dialysis and those who will not within this time frame, a minimum of 60 patients who commence dialysis within one year is required based on the calculation of 6 events per predictor variable (6 × 10).

## 3. Results

### 3.1. Baseline Characteristics of the Patients

**Figure 1:**
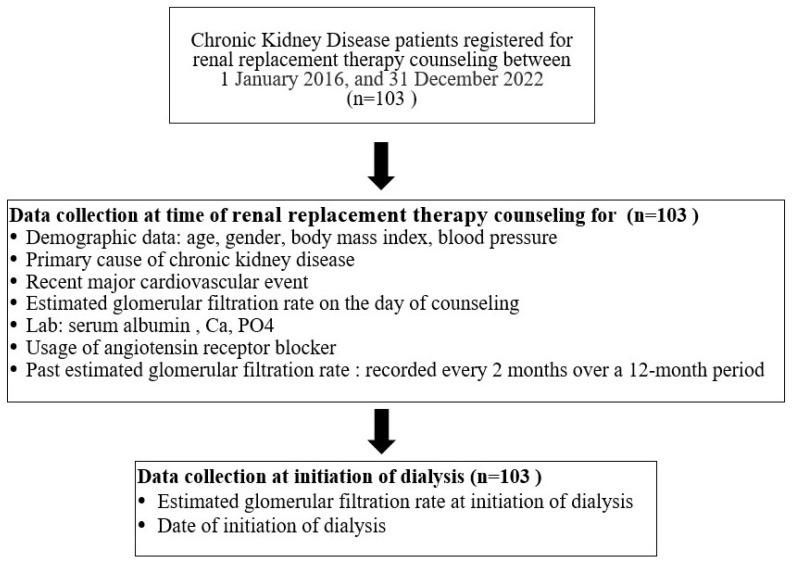
Study flow diagram.

**Figure 2:**
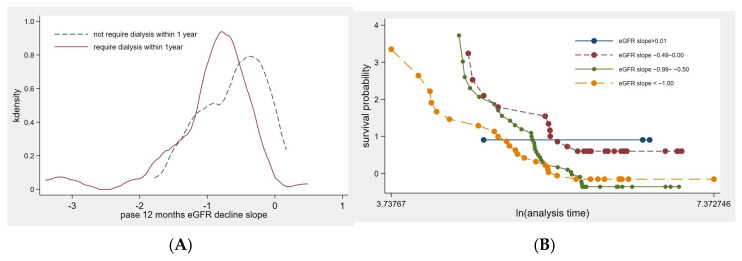
(**A**) Distribution of eGFR decline slopes in the study cohort, comparing patients with early KRT initiation to those without KRT initiation within one year. (**B**) Survival probability of different eGFR slope categories over the analysis period.

A total of 103 patients who enrolled in KRT counseling were included in this study (Figure 1). Of these, 62 patients required dialysis within one year of KRT counseling, while 2 patients died before initiating dialysis. The baseline characteristics of the study cohort are summarized in Table 1. The mean age of the participants was 63 years. Patients requiring early KRT initiation were, on average, younger than those who did not require dialysis within one year. Diabetes mellitus was the primary underlying cause of CKD, accounting for 63.1% of cases. Notably, 83% of patients with glomerular disease required dialysis within one year of KRT counseling. The mean eGFRi was 11.02 mL/min/1.73 m^2^. Patients who required dialysis received KRT counseling at a mean eGFRi of 10.74 mL/min/1.73 m^2^, while those who did not require dialysis within a year had a mean eGFRi of 11.31 mL/min/1.73 m^2^, with no significant difference observed between the two groups. The eGFR slope significantly differed between patients who initiated KRT within one year and those who did not (Table 1 and Figure 2A). At the time of the first dialysis session, the mean eGFR was 5.67 mL/min/1.73 m^2^.

### 3.2. Factors Influencing the Rate of eGFR Decline

The eGFRr ranged from −3.40 to 0.49 mL/min/1.73 m^2^/month, with only four patients (3.88%) showing a positive GFR slope. Factors influencing the eGFRr over the preceding twelve months were examined using Gaussian regression. This analysis identified age, severe hypoalbuminemia, serum calcium level, serum phosphate level at the time of KRT counseling, and recent use of RAASi as significant predictors of eGFRr (Table 2). Additionally, eGFRr was significantly associated with the initiation of dialysis within one year (Table 3).

### 3.3. Factors Associated with the KRT Initiation

**Figure 3:**
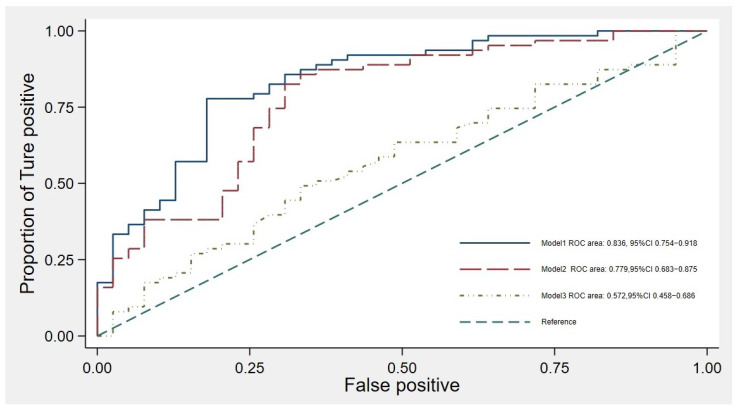
Area under the receiver operating characteristic (AuROC) curve for the prognostic prediction models. Model 1: Based on past eGFR decline rate (eGFRr), age, systolic blood pressure, BMI categories, primary cause of CKD, and serum calcium as predictors. Model 2: Based on age, systolic blood pressure, BMI categories, primary cause of CKD, and serum calcium as predictors. Model 3: Based on eGFR at the time of KRT counseling as a single predictor.

To differentiate between patients who would require dialysis within one year and those who would not, we incorporated eGFRr and five relevant clinical parameters into a predictive model. This model demonstrated an area under the receiver operating characteristic curve (AuROC) of 0.836 (95% CI 0.754–0.918) when assessed using binary logistic regression. The eGFRr-based predictive model exhibited superior discriminative performance in comparison to existing methods, such as the use of a single eGFR measurement at the time of KRT counseling (AuROC 0.572; 95% CI 0.458–0.686) or a combination of clinical parameters (AuROC 0.779; 95% CI 0.683–0.875) (Figure 2). The calibration of the model, as depicted in the calibration plot (Figure 3), compares the predicted probabilities with the observed probability of dialysis initiation within two years. In internal validation, employing bootstrap sampling, the model yielded an AuROC of 0.767 (95% CI 0.679–0.857), with model optimism measured at 0.069. The shrinkage factor was estimated to be 0.618.

### 3.4. Model Accuracy in the Study Cohort

**Figure 4:**
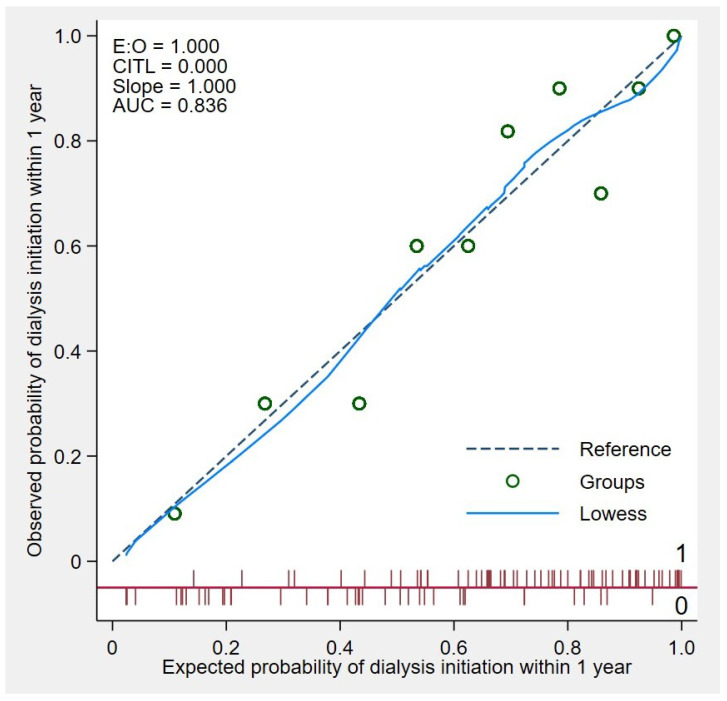
Calibration plot of the agreement between predictive model-predicted probability and observed probability of dialysis initiation.

Calibration plot showing the agreement between the predictive model-predicted probability and the observed probability of dialysis initiation (Figure 4). The plot evaluates the accuracy of the model by comparing predicted probabilities with actual outcomes within the study cohort.

## 4. Discussion

This study highlights the utility of assessing the 12-month eGFR trajectory prior to KRT counseling. Early initiation of KRT within one year was associated with younger age, lower serum albumin levels, hypocalcemia, and a more significant decline in total eGFR compared to patients who did not commence dialysis within the same timeframe (Table 1). Notably, the eGFRr was the sole factor that significantly influenced KRT initiation within one year (Table 3). Additionally, factors such as age, hypoalbuminemia, hyperphosphatemia, elevated calcium levels, and the current use of RAASi were associated with the eGFRr decline over the one-year period (Table 2). The 1-year eGFRr slope, along with clinical parameters, including age, systolic blood pressure, body mass index, cause of CKD, and serum albumin, demonstrated the highest AuROC for predicting KRT initiation (Figure 3). These findings suggest that a comprehensive evaluation of eGFR trends, combined with relevant clinical parameters, can enhance the prediction of dialysis initiation, thereby facilitating timely and individualized KRT planning.

Previous studies have shown that baseline eGFR levels can temporarily predict KRT initiation at one-year intervals over a three-year period when adjusting for age, sex, race, and diabetes among adults with an eGFR of 10 to 13 mL/min/1.73 m^2^, with an adjusted odds ratio of 1.053 (95% CI, 1.008–1.100) [9]. Our study primarily included patients with very advanced CKD due to our dialysis counseling policy, which advises SDM for patients with eGFR < 20 mL/min/1.73 m^2^. This approach results in a more homogeneous eGFR distribution, reducing potential variability caused by different CKD stages. However, our study demonstrated an average eGFR of 11.02 mL/min/1.73 m^2^ with a higher rate of KRT initiation within one year. Additionally, baseline eGFR did not predict KRT initiation in our study. The timing of dialysis initiation in CKD G5 patients is challenging. The optimal timing varies, and there is no consensus in the current KDOQI guidelines [3]. In the IDEAL study, randomized controlled trials comparing early KRT initiation (eGFR of 10–14 mL/min/1.73 m^2^; *n* = 404) versus late KRT initiation (eGFR of 5–7 mL/min/1.73 m^2^; *n* = 424) did not show any significant differences in death, cardiovascular events, or dialysis complications [7]. Our study demonstrated a mean eGFR at initiation of 5.67 mL/min/1.73 m^2^. Additionally, current registry data show that the average predialysis eGFR at initiation varies among countries (5 mL/min/1.73 m^2^ in Taiwan, 6.4 in New Zealand, 8.5 in the UK, 7.3 in Australia, 9–10 in Canada and France, and 11 in the US) [10]. In our study, we excluded patients with hemodynamic instability at KRT initiation as incremental hemodialysis is not suitable for this population. However, incremental hemodialysis, particularly twice-weekly sessions, is commonly performed in our country due to limited resources and the goal of preserving residual kidney function [11]. Decisions regarding dialysis initiation should involve shared decision-making with patients and be tailored to each individual’s circumstances during KRT counseling. Personalized and timely initiation of KRT is recommended to optimize patient outcomes, taking into account factors such as disease progression, patient preferences, and overall health status [7]. Risk predictions for KRT initiation have been reported for CKD stages G3–G4 within five years, including factors such as age, sex, eGFR, hemoglobin, proteinuria/albuminuria, systolic blood pressure, antihypertensive medication use, diabetes, and its complications [10,12]. In addition, early KRT initiation is associated with six-month mortality, with predictors including older age, comorbidities (especially coronary heart disease), underlying kidney disease, and late referral to nephrologists [10,13]. A systematic review and meta-analysis of 14 cohorts with almost 3.8 million participants demonstrated that a rapidly declining 3-year eGFR slope in early-stage CKD (stage ≥ 3b) predicted ESKD within five years in 8.3% of cases [14]. We also demonstrated that in CKD G5 patients at the time of counseling, a prior 12-month eGFR decline rate predicted KRT initiation. Early KRT initiation was associated with an average eGFR decline rate of −0.95 (0.67) mL/min/1.73 m^2^ per month, which was significantly different from those who did not initiate KRT within one year. Although eGFR trajectories do not always show a decline in some patients, our study included four patients with increasing eGFR (Figure 2B), while the eGFR slope remained stable or declined over time in most patients, as also demonstrated in the literature [15,16]. A rapid decline of eGFR exceeding 5 mL/min/1.73 m^2^ per year is associated with CKD progression [17]. Furthermore, an eGFR slope steeper than −10 mL/min/1.73 m^2^ per year necessitates timely arteriovenous fistula preparation [18]. The eGFR slope is generally recommended as a surrogate endpoint for CKD clinical trials in the literature and should be tailored to different kidney diseases [19,20]. Additionally, the eGFR slope is not only associated with CKD progression and ESKD but also with mortality and the timely creation of arteriovenous fistulas [10,14,18,21].

A cohort study in Japan involving 61,985 CKD G3 patients found that only 9.2% of subjects exhibited a rapid eGFR decline (faster than −2.0 mL/min/1.73 m^2^ per year) compared to those with a slower decline [22]. However, this study did not identify factors associated with the eGFR slope in diabetic patients, such as age, gender, baseline eGFR, HbA1c, and systolic blood pressure [22]. Hypertension is a well-recognized risk factor for CKD progression, as demonstrated in previous studies, particularly in patients with CKD stage ≥ 3b [23]. However, in our study, blood pressure did not significantly influence eGFR decline or dialysis onset within one year. Another cohort study demonstrated an inverse relationship between albuminuria and the improvement of eGFR slope during follow-up [16]. Our study demonstrates that the 12-month eGFRr since KRT counseling in CKD G5 patients is associated with KRT initiation. The factors that significantly accelerated eGFR decline include age, severe hypoalbuminemia, increased serum calcium levels, increased serum phosphate levels at the time of KRT counseling, and recent use of RAASi. However, RAASi was used in only eight patients in our study, which may introduce bias in interpretation. In our practice, RAASi was withdrawn in CKD G5 patients if they experienced hyperkalemia or accelerated eGFR decline over time. In contrast, other studies have shown that although RAASi use is associated with an acute eGFR drop, it ultimately contributes to a slower rate of renal function decline over time. Specifically, RAASi use has been linked to slower renal function decline in CKD G3b patients [24]. A meta-analysis of 22 cohorts demonstrated that an eGFR slope of −6 mL/min per 1.73 m^2^ per year compared to 0 mL/min per 1.73 m^2^ per year over the previous three years was associated with an adjusted hazard ratio of 2.28 (95% confidence interval, 1.88 to 2.76) for ESKD. Past eGFR decline is associated with future ESKD, especially when the eGFR is below 30 mL/min/1.73 m^2^ [25]. Our study added on the benefit of 12-month eGFR trajectory at the time of counseling KRT associated with the KRT initiation with in a year and factors associated with the eGFR slope in the very advance stage of CKD (CKD G5). Furthermore, the eGFR slope integrated with parameters such as age, systolic blood pressure, BMI categories, primary cause of CKD, and serum calcium has the highest performance of KRT initiation prediction, with an AuROC of 0.836 (95% CI of 0.754 to 0.918). Current clinical scoring systems for predicting the initiation of KRT, such as the kidney failure risk equation (KFRE-2), incorporate the urinary albumin–creatinine ratio and eGFR measured at the index clinic visit [26]. A KFRE-2 cutoff of greater than 10% is associated with KRT initiation within two years, with specificity increasing from 52% to 80% when the cutoff is adjusted to 30% [26]. In our study, however, we lacked quantitative albuminuria data due to the advanced stage of CKD during follow-up. Instead, we focused on factors such as the decline in eGFR, electrolyte abnormalities, and the management of CKD-mineral and bone disorder as critical predictors for KRT initiation. Notably, we were limited to using albumin dipstick measurements, which restricted our ability to quantitatively assess the impact of albuminuria.

One limitation of our study is the late-stage initiation of KRT counseling, which does not align with current guidelines. Despite this, our findings demonstrate the usefulness of the eGFR slope in predicting KRT initiation. A second limitation is the relatively small sample size; however, according to TRIPOD recommendations, our sample size was adequate for distinguishing between early KRT initiation within a year and patients who did not proceed to dialysis. Additionally, future studies should incorporate quantitative measures of albuminuria and the use of RAAS inhibitors or newer medications such as SGLT2 inhibitors and nsMRAs to enhance the prediction of eGFR slope. The applicability of our predictive model in CKD patients experiencing acute exacerbations due to infection, such as during the COVID-19 pandemic, warrants further investigation. In addition, our data limited the external validation; further prospective studies should use the predictive model to assess the rate of avoidance of catheter-dependent dialysis. Lastly, our study was limited by incomplete data on certain clinical parameters.

## 5. Conclusions

This study underscores the significant role of the 12-month eGFR trajectory in predicting the initiation of KRT within one year among patients with advanced chronic kidney disease (CKD G5). Our findings reveal that a more pronounced decline in eGFR, alongside factors such as younger age, lower serum albumin levels, hypocalcemia, and effective management of electrolyte abnormalities, serves as a robust predictor for early KRT initiation. Notably, the eGFR slope emerged as the sole significant determinant for KRT initiation within the specified timeframe, and when integrated with clinical parameters like age, systolic blood pressure, body mass index, primary cause of CKD, and serum calcium, the predictive model achieved a high AuROC. This study contributes to the growing evidence that dynamic monitoring of kidney function, particularly through eGFR slope analysis, provides a more accurate and actionable approach to anticipating the progression to end-stage kidney disease, thereby facilitating better-informed clinical decision-making and individualized patient care.

## Figures and Tables

**Table 1:** Baseline characteristics of the study patients.

Clinical Characteristics	Early KRT Initiation with in a Year (*n* = 64)	No KRT Initiation Within a Year (*n* = 39)	*p*-Value
Age, years, mean (SD)	60.97 (12.38)	67.21 (9.54)	0.008
Gender, male, *n* (%)	40 (36%)	23 (63%)	0.835
Body mass index, kg/m^2^, mean (SD)	25.41 (4.57)	25.05 (4.03)	0.709
Systolic blood pressure, mmHg, *n* (%)			0.031
<120	3 (60%)	2 (40%)	
120–139	15 (43%)	20 (57%)	
140–159	23 (72%)	9 (28%)	
≥160	23 (74%)	8 (26%)	
Causes of ESKD, *n* (%)			0.617
Diabetes mellitus	42 (65%)	23 (35%)	
Hypertension	10 (53%)	9 (47%)	
Glomerular disease	5 (83%)	1 (17%)	
PCKD	2 (67%)	1 (33%)	
Tubulointerstitial disease	1 (50%)	1 (50%)	
Others	4 (50%)	4 (50%)	
Mode of KRT			0.586
Peritoneal dialysis	12 (70.59)	5 (29.41)	
Hemodialysis	52 (60.47)	34 (39.53)	
History of CV events, *n* (%)	5 (71%)	2 (28%)	0.707
RAASi, *n* (%)	8 (100%)	0 (0%)	0.019
Hemoglobin, g/dL, mean (SD)	10.00 (1.48)	10.38 (1.56)	0.215
Serum albumin, g/dL, mean (SD)	3.66 (0.55)	3.88 (0.46)	0.035
Serum phosphate, mg/dL, mean (SD)	4.33 (0.88)	4.12 (0.67)	0.218
Serum calcium, mg/dL, mean (SD)	8.66 (0.62)	9.06 (0.58)	0.001
eGFRc, mL/min/1.73 m^2^, mean (SD)	10.84 (2.21)	11.31 (2.29)	0.304
eGFRi, mL/min/1.73 m^2^, mean (SD)	5.67 (1.7)	-	-
Average rate of eGFR decline, mL/min/1.73 m^2^/month, mean (SD)	−0.95 (0.67)	−0.62 (0.48)	0.007

Abbreviations: CV events, cardiovascular events; eGFRc, eGFR at the time of KRT counseling; eGFRi, eGFR at the time of the first dialysis session; ESKD, end-stage kidney disease; PCKD, polycystic kidney disease; RAASi, renin–angiotensin–aldosterone inhibitors.

**Table 2:** Factors influencing the rate of GFR decline over the past twelve months preceding KRT counseling (eGFRr).

Variables	Coefficient (SE)	95% Confident Intervals	*p*-Value
Age	0.013 (0.006)	0.002 to 0.024	0.019
Serum albumin < 2.5 mg/dL	−1.157 (0.243)	−1.633 to −0.680	<0.001
Serum calcium level	−0.330 (0.104)	−0.535 to −0.125	0.002
Serum phosphate > 4.5 mg/dL	−0.405 (0.135)	−0.669 to −0.141	0.003
RAASi	−0.741 (0.208)	−0.150 to −0.333	<0.001

Abbreviation: RAASi, renin–angiotensin–aldosterone inhibitors.

**Table 3:** Factors influence on the dialysis onset within 1 year.

Variable	SHR (SE)	95% Confident Intervals	*p*-Value
eGFRr, mL/min/1.73 m^2^/month	0.656 (0.123)	0.454 to 0.948	0.025
eGFRi, mL/min/1.73 m^2^	0.995 (0.003)	0.989 to 1.001	0.132
Age < 70 year	1.620 (0.639)	0.748 to 3.509	0.221
Male	1.482 (0.402)	0.871 to 2.521	0.146
Body mass index, kg/m^2^			
18.50–24.99	2.056 (2.021)	0.300 to 14.108	0.463
25.00–29.99	0.894 (1.902)	0.265 to 13.547	0.524
≥30	12.060 (1.205)	0.253 to 16.788	0.499
Systolic blood pressure, mmHg			
120–139	0.632 (0.553)	0.114 to 3.514	0.600
140–159	1.578 (1.382)	0.284 to 8.782	0.602
≥160	2.588 (2.402)	0.412 to 15.960	0.306
Diabetic nephropathy	0.726 (0.314)	0.311 to 1.694	0.459
Major cardiovascular events	1.685 (0.921)	0.577 to 4.916	0.340
RAASi	0.940 (0.323)	0.480 to 1.842	0.858
Serum albumin, mg/dL	0.947 (0.039)	0.874 to 1.025	0.179
Serum calcium, mg/dL	0.969 (0.019)	0.932 to 1.007	0.111
Serum phosphate, mg/dL	0.977 (0.019)	0.941 to 1.014	0.224

Abbreviation: eGFRi, eGFR at the time of the first dialysis session; RAASi, renin–angiotensin–aldosterone inhibitors; SHR, subdistribution hazard ratio.

## Data Availability

The datasets used and/or analyzed in the current study are available from the corresponding author upon reasonable request.

## Abbreviations

The following abbreviations are used in this manuscript:

AuROC	Area under the receiver operating characteristic curve
BMI	Body mass index
CKD	Chronic kidney disease
ESKD	End-stage kidney disease
eGFR	Estimated glomerular filtration rate
eGFRr	Rate of change in eGFR over the 12 months preceding the first KRT counseling session
eGFRi	eGFR at the time of the first dialysis initiation
KRT	Kidney replacement therapy
KFRE-2	Kidney failure risk equation
RAASi	Renin–angiotensin–aldosterone system inhibitors
LOWESS	Locally weighted scatterplot smoothing
TRIPOD	Transparent reporting of a multivariable prediction model for individual prognosis or diagnosis
